# A Retrospective Cross-Sectional Cohort Trial Assessing the Prevalence of MTHFR Polymorphisms and the Influence of Diet on Platinum Resistance in Ovarian Cancer Patients

**DOI:** 10.3390/cancers13205215

**Published:** 2021-10-18

**Authors:** Caitlin Phillips-Chavez, Jermaine Coward, Michael Watson, Janet Schloss

**Affiliations:** 1 Icon Cancer Centre, Queensland, Australia; jim.coward@gmail.com; 2 Endeavour College of Natural Health, Brisbane, QLD 4006, Australia; higher_primate1968@yahoo.com.au; 3 School of Medicine, University of Queensland, Brisbane, QLD 4006, Australia; 4 Institute of Health & Biomedical Innovation, Queensland University of Technology, Brisbane, QLD 4006, Australia; 5 NCNM, Southern Cross University, Lismore, NSW 2480, Australia; janet.schloss@scu.edu.au

**Keywords:** platinum resistance, methylation, epithelial ovarian cancer, methylene tetrahydrofolate

## Abstract

**Simple Summary:**

Ovarian cancer (EOC) has a very poor prognosis, with a 5-year survival rate of just 43%. One of the biggest challenges is the resistance to standard chemotherapeutics. Nutrition modification is a potential adjunct that may be suitable to support cancer therapies through epigenetic modifications of DNA and biochemical pathways associated with drug response. It was retrospectively hypothesised that carrying a methylenetetrahydrofolate reductase (MTHFR) gene polymorphism may affect chemo response in EOC, and that nutrient status may further influence response to standard platinum therapy. This small pilot study of twenty-five participants provided a novel foundation for identifying that dietary intake of vitamins B12, B6 and zinc may impact platinum-drug response in EOC dependent on MTHFR genotype. Further large-scale investigations are required to verify the findings of this study.

**Abstract:**

Ovarian cancer has the lowest survival rate in gynaecologic malignancies with a 5-year survival rate of 43%. Platinum resistance is one of the main drivers of ovarian cancer mortality, of which aberrant methylation has been cited as a significant contributor. Understanding the essential role of the methylenetetrahydrofolate reductase enzyme (MTHFR) on DNA synthesis and repair, and how nutrient status can vastly affect its performance, led to the investigation of MTHFR status and dietary influence on platinum response in epithelial ovarian cancer (EOC) patients. Twenty-five adult female patients who completed first-line platinum-based chemotherapy for primary ovarian cancer were selected from Icon Cancer Centres in Australia. Participants were grouped based on platinum response. A full medical and family history, food frequency questionnaire and single blood test were completed, testing for MTHFR polymorphisms, serum folate, serum and active B12 and homocysteine levels. Nineteen of twenty-five participants had an MTHFR polymorphism. Of those, 20% were compound heterozygous, 12% were heterozygous C677T (CT), 4% homozygous C677T, 12% homozygous A1298C and 28% were heterozygous A1298C (AC). Statistically significant associations were found between dietary zinc (*p* = 0.0086; 0.0030; 0.0189) and B12 intakes in CT genotypes (*p* = 0.0157; 0.0030; 0.0068) indicating that zinc or vitamin B12 intakes below RDI were associated with this genotype. There were strong associations of vitamin B6 intakes in AC genotypes (*p* = 0.0597; 0.0547; 0.0610), and dietary folate in compound heterozygotes with sensitive and partially sensitive disease (*p* = 0.0627; 0.0510). There were also significant associations between serum folate (*p* = 0.0478) and dietary B12 (*p* = 0.0350) intakes above RDI and platinum sensitivity in wild-types as well as strong associations with homocysteine levels (*p* = 0.0886) and zinc intake (*p* = 0.0514). Associations with dietary B12 (*p* = 0.0514) and zinc intakes (*p* = 0.0731) were also strong in resistant wild types. Results indicate that dietary zinc, B12 and B6 intakes may be associated with platinum sensitivity dependent on MTHFR genotype. These results require further research to clarify the dosages necessary to elicit a response; however, they provide a novel foundation for acknowledging the role of diet on treatment response in EOC.

## 1. Introduction

Epithelial ovarian cancer (EOC) is a highly heterogenous disease with its subtypes reported to share very few molecular similarities [1]. It is also considered the most lethal gynaecological malignancy, representing the eighth most common cause of all types of cancer deaths in women worldwide [2]. Although ovarian cancer is deemed highly curable if diagnosed in its early stages, over 75% of patients present with stage III/IV disease which garners a 5-year survival rate of just 43% [3]. Initial management commonly revolves around aggressive cytoreductive surgery, which is either preceded or followed by chemotherapy containing a platinum and taxane doublet with the addition of vascular endothelial growth factor antagonist (VEGF) [4] if optimal debulking is not achieved. The success of debulking surgery has been considered a valuable prognostic variable and research indicates that the eradication of microscopic disease may improve patient outcomes [5,6]. However, the development of platinum resistance continues to be a significant clinical challenge since the introduction of platinum therapy in the 1970s [4]. A multitude of pathways and processes have been identified as contributing to the development of platinum resistance in EOC, including reduced drug influx, increased drug efflux and competent DNA repair pathways and, most recently, aberrant methylation [7,8,9]. The aim of this study was to investigate the prevalence of gene polymorphisms in a key methylation pathway, and dietary influence on drug response in ovarian cancer patients.

Platinum response rates fall on a continuum resulting in the development of four classifications of recurrent disease defined by the time elapsed between recurrence and last exposure to platinum-based chemotherapy, i.e., platinum free interval (PFI). Namely platinum-refractory (<1 month), platinum-resistant (1–6 months), partially platinum-sensitive (6–12 months), and platinum-sensitive (>12 months) disease as defined by the Gynaecological Cancer InterGroup (GCIG) [10,11]. Increasing evidence suggests oncologic signalling is linked to metabolic rearrangements enabling cancer cell proliferation, including deregulations of one-carbon (1C) metabolism, involving three key pathways: folate, methionine and transsulphuration pathways [12]. EOC cells in particular have been reported to be characterised by a cholinic phenotype and the overexpression of folate receptor alpha (FRα), which has a higher affinity to synthetic folic acid [12,13,14]. Folic acid has an extremely low rate of conversion to dihydrofolate (DHF) compared to its natural folate counterpart and research indicates there may be a relationship with high circulating unmetabolised folic acid and tumorigenesis. Low concentrations of DHF diminish the tetrahydrofolate (THF) pool, which in turn alters the concentration of 5-methyltetrahydrofolate (5-MTHF) that may inhibit Glycine N-methyltransferase (GNMT), altering the S-adenosyl methionine (SAM) and S-adenosyl homocysteine (SAH) ratio, potentially impacting DNA methylation [15,16]. Furthermore, increased intracellular concentrations of glutathione (GSH) and metallothionein (MT) have been implicated in the development of drug resistance in ovarian cancer, and are strongly linked to the methylation cycle through its supply of substrate cysteine to the transsulphuration pathway [17,18].

The methylation cycle is a significant and essential biochemical pathway that is dependent on an adequate supply of cofactors, folate (vitamin B9), vitamin B6, B12, choline and serine to function [19]. The methylation cycle is dependent on the adequate functioning of a number of key enzymes. One of particular interest is methylene tetrahydrofolate reductase (MTHFR) which is responsible for the reduction of folate into its metabolically active form. MTHFR is crucial in catalysing the conversion of folate to 5-methyltetrahydrofolate (5-MTHF), homocysteine to methionine and contributing to the production of MT, GSH and essential methyl donor SAM [20]. The rate at which the MTHFR enzyme functions is determined by a number of different factors, including the type of polymorphism present (Table 1). Some medications such as neuropsychotropics, anticonvulsants, hormone replacement therapy (HRT) and lipid lowering drugs may be pharmacogenetically influenced by MTHFR polymorphisms through the interaction with folate and homocysteine metabolism and the depletion of labile methyl groups, altering drug response rate and potentiating toxicities [21].

A number of studies have identified that improper or impeded methylation of the MTHFR gene can result in diminished MTHFR enzyme activity. This reduction in enzyme activity has been linked with general impairment of DNA methylation within cells, which has been found to lead to disease development, including sperm dysfunction [23,24].

Methylation of cytosine phosphate guanine (CpG) islands has been identified as a key player in platinum resistance in EOC with a high incidence of hypermethylated genes, and to a lesser extent, hypomethylated genes [25]. Zhang [26] suggests that there are three ways in which nutrition influences DNA methylation. Firstly, through the provision of substrates necessary for DNA methylation; second, the provision of cofactors that assist in the modulation of DNA methyltransferase, whose enzyme activities are responsible for the addition of methyl groups de novo and the copying of methylation patterns during cell replication and thirdly, by changing the activity of enzymes responsible for 1C metabolism, one of the most investigated and most related to this study being MTHFR.

Folate status is closely related to the functioning of the methylation cycle, as it is the initiating nutrient within the cycle [27]. However, due to dependence on substrates such as vitamin B6 and B12 for the adequate function of the methylation cycle, our study aimed at investigating other nutrients to support the understanding of the complex synergism within the methylation cycle and associated biochemical pathways [28]. Previous research shows that even mild folate restriction results in drastically reduced methionine levels, which significantly decreases the cellular SAM to SAH ratio despite the percentage of 5-methyl deoxycytidine in genomic DNA remaining unchanged. On single strands of DNA, 5-methyl deoxycytidine act as a *cis* signal for de novo methylation, indicating that there is another signalling pathway associated with DNA methylation. Furthermore, mild folate restriction appears to have stronger impacts on purine synthesis in homozygous C677T (TT) carriers than homozygous A1298C (CC) and overall homocysteine remethylation. When folate levels are adequate, the MTHFR genotype does not appear to affect remethylation [29]. Conflicting evidence on the effect of folate on DNA methylation demonstrates a more complex process than traditionally thought, one which continues to be investigated.

Metallothionein (MT) has also been implicated in the development of drug resistance in ovarian cancer cells through the direct chelation of platinum through thiol groups on cysteine molecules providing cell protection and supporting detoxification [30,31,32]. MT also moderates the supply of zinc during G1/S cell cycle transition. This impacts the function of zinc-dependent transcription factors during which time the cell contents and chromosomes are replicated, potentiating the possibility of disordered replication [18]. Zinc is also an essential nutrient for p53 tumour suppressor activity, responsible for appropriately induced apoptosis. MT have been reported to remove zinc from p53 proteins leading to changes in structure and ultimately inactivation, however the direct interaction between MT and p53 in cells is not fully understood [33]. Inactivation of p53 may result in uncontrolled cell proliferation and tumorigenesis [18,30].

Currently there are no recommended changes in clinical management of patients found with an MTHFR gene variant, however in this study we aim to assess whether there may be any association with platinum response, dietary intakes of methylation cycle substrates zinc, B6, B12 and folate and MTHFR polymorphisms [34].

## 2. Materials and Methods

This non-randomised observational pilot trial was conducted across Icon Cancer Centre sites in Queensland, Australia between August and December 2018. Ethical approval was provided through Endeavour College of Natural Health Human Research Ethics Committee (HREC No. 20180609) with a written reciprocal agreement with Icon Cancer Centre. The trial was registered with the Australian and New Zealand Clinical Trials Registry (ANZCTR12618001138279). Inclusion criteria included female adults, aged 18 to 80, who had been treated with a platinum-based regime for primary EOC, fallopian tube or peritoneal cancers and were a minimum of six months post first-line treatment.

### 2.1. Data Collection

A case report form (CRF) focused on patient demographics, including medical history, current treatment regime, platinum response status and other relevant information. A blood pathology test was conducted by Sullivan Nicolaides blood pathology by a trained phlebotomist after consent and baseline visit for MTHFR polymorphism, homocysteine, B12 and folate to determine functional competency of nutrient utilisation and methylation cycle. The blood pathology was taken on fasting and with only one time point. Participants whose serum B12 measured below 360 pmol/L or less (*n* = 14; 56%) were further tested for levels of holotranscobalamin, or ‘active B12’ (as per pathology centre guidelines). A diet history was collected via validated assessment tool, the Dietary Questionnaire of Epidemiological Studies version 3.2 (DQES_v3.2^®^) from the Cancer Council Victoria [35].

MTHFR analysis was based on three criteria: (1) presence of polymorphism by blood pathology testing; (2) single nucleotide polymorphism (SNP) variation—heterozygous or homozygous and (3) call letter and location variation.

Quality of life (QoL) was assessed using the Functional Assessment of Cancer Therapy—Ovarian Cancer (FACT-O^®^) [36] tool to quantify how treatment has affected the target population. Previous research highlights the impact of side effects related to diagnosis and treatment on well-being and overall functioning [37].

### 2.2. Data Analysis

Data analysis was performed on STATAv14 software. Associations between the presence or absence of MTHFR polymorphisms across the samples, nutrient intakes and blood serum markers were subject to the chi-square (χ^2^) test and Fisher’s exact (FE) tests due to small sample size. Logistic regression measures were used to assess blood pathology markers with smoking status, recreational drug use and alcohol intake. Logistic regression was also used to assess tumour type, platinum response, blood serum markers against the presence of MTHFR polymorphisms. Multivariate logistic regression was applied to determine the correlation between the existence of MTHFR polymorphisms, in relation to platinum response, blood serum markers and dietary intake of vitamins B6, B12, zinc and folate, identifying whether any of the variables can be used as a predictive measure for chemo response. All statistical significance was set at α = 0.05 with a power of 80%.

## 3. Results

### 3.1. Participant Characteristics

**Figure 1:**
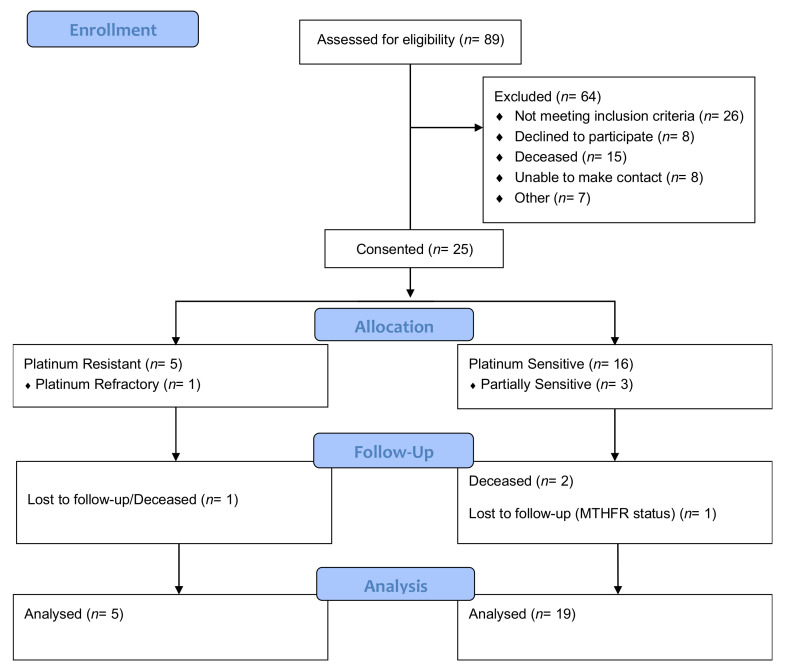
Consort flow diagram: participant inclusion and exclusion at each stage of the trial.

A total of eighty-nine ovarian cancer patients were identified. Twenty-five participants (*n* = 25) were consented for the trial. Sixty-four patients were excluded due to not meeting the inclusion criteria (*n* = 26), being unable to be contacted (*n* = 8), declining to participate (*n* = 8), being deceased (*n* = 15) and for ‘other’ reasons (*n* = 7) (Figure 1).

The participants’ median age was 64 years (range 41–78 years), median height 161.8 cm (±1.8 standard deviation [SD]), median weight 72.3 kg (SD ± 5.5), BMI in the overweight range of 27.47 (SD ± 1.96) and waist circumference median of 96 cm (SD ± 4.2) putting participants in the ‘higher risk’ range for chronic disease [38]. Confounders reported for these measurements included the presence of hernia and non-excised tumour exacerbating readings.

### 3.2. Tumour and Treatment Characteristics

The study participants received treatment for epithelial ovarian cancer (*n* = 14; 56%), primary peritoneal cancer (*n* = 7; 28%) or fallopian tube cancer (*n* = 3; 12%). All participants had completed first-line platinum-based chemotherapy of various platinum, taxane, anthracycline, alkylating agent and radiotherapy regimens, as described in Table 2. Debulking was recorded as optimal (*n* = 8; 32%), suboptimal (*n* = 6; 24%), and undisclosed (*n* = 11; 44%). Patient tumour characteristics are outlined in Table 3.

### 3.3. Pathology Results and Genetic Testing

Mean serum folate (25.6 nmol/L ± 1.8; 95% CI 21.90, 29.34), serum B12 (401 pmol/L ± 58; 95% CI 281.08, 521. 16), active B12 (73 pmol/L ± 7.88; 95% CI 55.96, 90.03) and homocysteine (11.21 μmol/L ± 0.91; 95% CI 9.32, 13.10) all fell within normal reference ranges (11–57 nmol/L, 118–701 pmol/L, ≥42.48 pmol/L and 4–14 μmol/L, respectively). Participants’ lifestyle habits were assessed against their serum folate, homocysteine and B12 pathology, with no statistical significance found using logistic regression.

**Figure 2:**
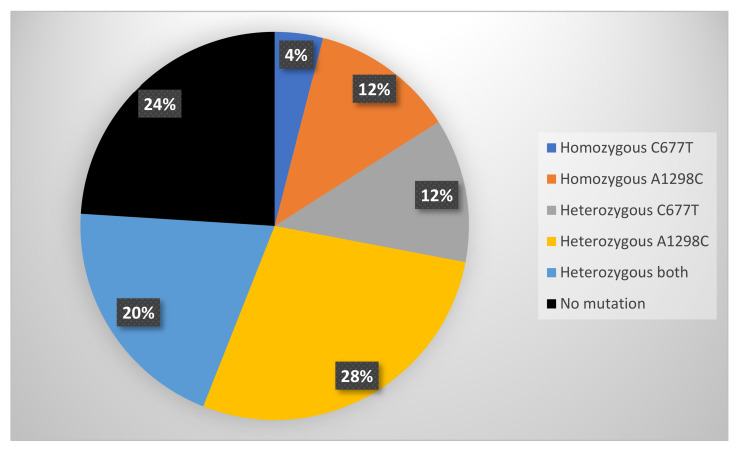
MTHFR mutations represented in the trial population: The trial population represented the entire range of C677T and A1298C MTHFR mutations, including a high proportion of compound heterozygous mutations.

Seventy-six percent of participants (*n* = 19) tested positive for a polymorphism. MTHFR distribution within the study as illustrated in Figure 2.

### 3.4. Intergroup Analysis

No statistical significance was noted on MTHFR polymorphisms and serum blood results. A relationship between serum homocysteine level and TT mutation trended towards statistical significance (*p* = 0.068; −0.42–0.01 CI) (Table 4). Active B12 levels and TT results were nearing significance (χ^2^ *p* = 0.055). Serum B12 levels without a polymorphism were also trending towards statistical significance with two participants falling above reference range and two below (χ^2^ *p* = 0.062), however this association was not seen using FE tests (Table 5).

Multivariate analysis revealed no statistically significant results between the presence of a polymorphism and platinum response. Furthermore, logistic regression revealed no statistically significant results between the presence of an MTHFR mutation and cancer type.

### 3.5. Dietary Analysis

FE test and χ^2^ analysis were applied to dietary intakes above and below recommended daily intake (RDI) levels and MTHFR polymorphism. Of statistical significance were the associations between dietary B12 and zinc intakes below RDI and participants with CT polymorphisms (χ^2^ *p* = 0.007; FE *p* = 0.024). AC polymorphisms were also found to have a strong association with dietary B6 intake (χ^2^ *p* = 0.018), although only trending towards statistical significance by the FE test (*p* = 0.070). All other tests were insignificant (Table 6). There were no significant changes reported in dietary habits or nutraceutical use prior to or throughout treatments.

For participants with no mutation (wild types), serum folate and dietary B12 intake were associated with platinum sensitivity (Pt-S) (*p* = 0.0478 and 0.0350) with serum homocysteine showing some relationship, however not statistically significant (*p* = 0.0886). The same participants saw relationships trending towards significance for dietary zinc intake for Pt-S and platinum resistance (Pt-R) (*p* = 0.0514 and 0.0731, respectively) and dietary B12 intake and Pt-R (*p* = 0.0514). CT genotypes were found to have statistical significance between vitamin B12 intake and Pt-S (*p* = 0.0157), partial sensitivity (*p* = 0.0030) and Pt-R (*p* = 0.0068). The same trend was seen between dietary zinc intakes and platinum response (*p* = 0.0086; 0.0030 and 0.0189, respectively). CT and folate intake were trending towards statistical significance and partial sensitivity (*p* = 0.0790). Dietary vitamin B6 intake and AC were also trending towards significance with Pt-S (*p* = 0.0597), partial sensitivity (*p* = 0.0547) and Pt-R (*p* = 0.0610). Furthermore, compound mutations were nearing significance between dietary folate intake, partial and complete Pt-S (*p* = 0.0510 and 0.0627, respectively) (Table 7).

## 4. Discussion

The sample population of this study was consistent with epidemiological data for age at diagnosis of EOC and BMI in the overweight range [39]. Furthermore, the study represented four of the five primary EOC histotypes with the inclusion of a Müllerian tumour which represents <2% of ovarian tumours in the literature [40,41]. Suboptimally debulked disease status traditionally predisposes patients to a poorer PFI [42], however amongst our population, this particular patient cohort demonstrated equal Pt-R and sensitive disease. This supports the notion that a variable number of molecular diversities are involved in each ovarian histotype, and that individuality in response to treatment cannot be purely explained by genetics alone [43].

This study cohort, although small, indicated an MTHFR prevalence much higher than those cited in the general population [34]. The C677T SNP is the most frequently investigated polymorphism, however the results of our study suggest that compound mutations may be higher in ovarian cancer patients, but further studies are required to confirm this finding.

Epigenetic modifications can occur throughout a person’s lifespan [44] which can affect the presentation of a person. One of the most extensively investigated modifiers is DNA methylation, which occurs within the 1C metabolic pathway. It is dependent on adequate cofactors such as folate, vitamins B6 and B12, as well as enzyme activity that includes the MTHFR enzyme [45]. In our study, dietary folate intakes indicated a trend towards a relationship with partial platinum sensitivity and CT genotypes, as well as with partial and complete sensitivity in compound heterozygotes. However, our results do not clearly indicate whether the dietary relationship is more strongly associated with folate intakes above or below RDI, requiring further research to identify adequate intake levels to augment optimal platinum response. If DNA methylation is potentially restricted in the presence of a mutation and inadequate dietary intakes of key nutrients such as folate, as suggested by previous literature, this may support the notion that hypomethylation in these patients may result in improved platinum response [26].

Previous research indicates that folic acid and B12 smentation in CC and TT genotypes with previous colorectal adenomas may potentially increase the risk of neoplastic transformations [46]. This is consistent with the data showing the overexpression of folate receptor alpha (FRα) in ovarian cancer cells, a receptor that has a higher affinity for folic acid, which allows tumour cells to proliferate even in low folate environments by transporting folic acid into cells [47]. Phase III trials on FRα targeting the antibody–drug conjugate Mirvetuximab soravtansine (IMGN853) are currently underway with positive preliminary results [48]. In addition to identifying folate receptors as a novel drug target, our study suggests a complementary strategy of manipulating diet to support primary treatment and may provide some grounds to investigate whether folate receptor expression is associated with MTHFR polymorphisms

In this study cohort, dietary zinc intakes were found to be significantly associated with platinum response in CT genotypes; however, the impact and dose requires further investigation. One possible mechanism that may explain our associations is that CT mutations in the presence of low zinc intake may lead to a reduction in MT. This may reduce the silencing of the p53 protein through alterations in zinc transfer reactions [33,49]. In wild types, however, a stronger association begins to emerge with zinc intakes that appear to be above RDI and Pt-S. This may be due to adequate zinc levels being available to the p53 protein to support appropriate programmed cell death or possibly a moderation of methylation, although further research is required to support either notion [49]. High concentrations of MT in chemotherapy-resistant osteosarcoma cells reported reduced intracellular zinc levels which may impact zinc-dependent p53 proteins.

Pathogenic sequences of the TP53 gene, which codes the p53 protein, have been found in 95.8% of HGSOC and an overexpression has also been found in endometrioid tumours [50,51]. In mucinous carcinomas, TP53 mutations are represented by a late event, in conflict to early events seen in HGSOC, suggesting an alternative role of p53 within this tumour type [43]. The research is conflicting on aberrant methylation in regards to p53. One study indicated that methylation in the promoter region of p53 may play a role in carcinogenesis of ovarian cancer, whereas another suggested that aberrant methylation was not associated with p53 mutations [52,53]. There appears to be a relationship between p53 expression and carcinogenesis of ovarian cancer, but it is unclear whether this is a result of methylation.

Nutrition advice surrounding cancer therapy is generally limited to ‘eating a healthy, balanced diet’ with cereals and grains which are often fortified with folate, legumes/beans and vegetables sharing an equal balance in the Australian Dietary Guidelines [54]. Low-fat dairy products, lean meats and fruits contribute equal parts of the remaining portions in the dietary guide. Furthermore, the primary dietary recommendations for patients during treatment is limited to energy requirements (kilojoules/calories) [55]. This pilot study shows that there may be a significant relationship with the types of nutrients the body is receiving and in what quantities, perhaps not only in terms of the cancer itself but also supporting adjuvant chemotherapy success. This study provides a novel example of how nutrients may potentially impact the response to first-line platinum drug therapy in epithelial ovarian cancer patients. However, larger studies are required to assess the impact of the preliminary findings.

A report formulated by the American Cancer Society suggests that nutritional screening and assessment should begin whilst treatment is being planned. It focuses primarily on avoiding malnutrition and associated comorbidities, rather than to specifically complement the treatment process: ‘During active cancer treatment, the overall goals of nutritional care for survivors should be to prevent or reverse nutrient deficiencies, to preserve lean body mass, to minimize nutrition-related side effects (such as decreased appetite, nausea, taste changes, or bowel changes), and to maximize quality of life’ [56]. The aim of our study was to identify whether nutritional status may impact platinum response in ovarian cancer patients, and may ultimately be employed to complement primary therapy to improve outcomes in a population that largely suffers poorer prognosis. From the preliminary results included herein, there may be some benefit in addressing nutrition from the perspective of improving platinum response through dietary manipulation, however, larger studies would be needed to substantiate this view.

There are a number of limitations to this study. Firstly, retrospective study designs, although traditionally less expensive, are able to be run over shorter time frames. Limitations in cohort studies commonly feature a lack of documentation or lost data [57]. This is particularly evident with the lack of data able to be obtained from medical records in our study. Secondly, sample size may be the most significant limitation in this study. This is particularly evident from the relationships seen between homocysteine levels and TT carriers, where the results were trending towards statistical significance. Similarly, the relationship that was beginning to emerge between smoking and serum folate levels did not have adequate power for a comprehensive outcome. Both results are consistent with the literature, that there are relationships between both, however the *p*-value is indicative of an underpowered study [58,59]. Furthermore, time constraints meant recruitment was limited in a population where hospitalisations and rapid disease progression are a common barrier.

## 5. Conclusions

The current study presents the first evidence, to the best of our knowledge, that improved response to platinum therapy in MTHFR wild types may be associated with dietary B12 intakes, and to a lesser extent, dietary zinc. Similarly, platinum response may be influenced by dietary B12 and zinc intakes in CT genotypes, and to a lesser extent, dietary B6 intakes in AC genotypes. Dietary folate intakes appear to influence platinum sensitivity in compound heterozygotes and that the presence of MTHFR polymorphisms may mediate the influence of dietary folate intakes. The dosages of nutrients required to elicit an effect on platinum response in EOC patients require further investigation. This study also shows that blood serum tests may be inadequate to determine a relationship between dietary intakes and response to therapy as a prognostic tool. Further genomic testing is required to adequately determine the extent of the relationship between the presence or absence of MTHFR polymorphisms and nutrient intakes on platinum response, and the potential impact on altered methylation signatures within tumour tissues.

## Figures and Tables

**Table 1:** Percentage of enzyme activity: percentage of normal MTHFR enzyme activity in the presence of different MTHFR gene mutations (*Adapted from:* Iverson Genetic Diagnostics Incorporated, 2016 [22]).

Genotype	Zygosity	% Normal Enzyme Activity
1298A/677C	−/− (Normal)	100% activity
A1298C	+/− (Heterozygous)	83% activity
C677T	+/− (Heterozygous)	66% activity
A1298C	+/+ (Homozygous)	61% activity
C677T/A1298C	Compound heterozygous	48% activity
C677T	+/+ (Homozygous)	24% activity

**Table 2:** Therapy Characteristics: Chemotherapy regimens in study participants.

Characteristics First-Line Therapy	Participants (*n* = )
IP cisplatin 75 mg/m^2^ and paclitaxel 135 mg/m^2^	1
Carboplatin AUC2 and paclitaxel 80 mg/m^2^	8
Carboplatin AUC5, Caelyx^®^ 30 mg/m^2^	1
Carboplatin AUC5, Caelyx^®^ 30 mg/m^2^ and Avastin^®^ 15 mg/m^2^	1
Carboplatin AUC5 and paclitaxel 175 mg/m^2^	2
Carboplatin and ifosphamide	1
Carboplatin AUC5 and docetaxel 75 mg/m^2^	2
Carboplatin AUC2 and paclitaxel 75 mg/m^2^	2
Carboplatin AUC2 and paclitaxel 64 mg/m^2^	1
Carboplatin and radiotherapy	1
Undisclosed carboplatin regime	5

**Table 3:** Tumour characteristics: tumour stage, grade, histotype, surgery and platinum response in study participants.

Characteristics: Type of Cancer	Participants (*n* = )
Ovarian	14
Peritoneal	7
Fallopian Tube	3
**Stage of Cancer**	**Participants (*n* = )**
Stage 1	1
Stage 1A	1
Stage 1C	2
Stage 2C	1
Stage 3	1
Stage 3C	8
Stage 4	6
**Tumour Histotypes**	**Participants (*n* = )**
Serous	18
Endometriod	3
Clear-cell	1
Mixed clear-cell and endometrioid	1
Mixed serous and endometrioid	1
Carcinosarcoma (Müllerian)	1
**Surgery**	**Participants (*n* = )**
Optimal debulking	8
Suboptimal debulking	6
Debulking status undisclosed	11
**Platinum Response**	**Participants (*n* = )**
Sensitive	16
Partially sensitive	3
Resistant	5
Refractory	1
**Tumour Grade**	**Participants (*n* = )**
High	22
Low	1
Undefined	2

**Table 4:** Serum pathology compared to MTHFR mutations: logistic regression was used to assess the relationship between individual MTHFR mutations and serum blood tests.

MTHFR Allele.	*p*-Value	Coefficient	Stand. Error	95% CI
**Serum Folate**				
Homozygous C677T	0.199	0.27	0.21	−0.14–0.7
Homozygous A1298C	0.53	0.05	0.08	−0.11–0.21
Heterozygous C677T	0.57	0.04	0.08	−0.11–0.21
Heterozygous A1298C	ND	ND	ND	ND
Heterozygous C677T + A1298C	0.76	−0.02	0.07	−0.17–0.12
**Serum Vitamin B12**				
Homozygous C677T	0.46	−0.005	0.003	−0.1–0.001
Homozygous A1298C	0.40	−0.001	0.001	−0.003–0.001
Heterozygous C677T	0.14	−0.002	0.001	−0.006–0.0009
Heterozygous A1298C	ND	ND	ND	ND
Heterozygous C677T + A1298C	0.29	−0.001	0.001	−0.004–0.001
**Homocysteine**				
Homozygous C677T	0.068	−0.02	0.11	−0.42–0.01
Homozygous A1298C	0.12	−0.09	0.06	−0.22–0.2
Heterozygous C677T	0.12	−0.09	0.06	−0.21–0.02
Heterozygous A1298C	ND	ND	ND	ND
Heterozygous C677T + A1298C	0.344	−0.044	0.04	−0.13–0.04

**Table 5:** MTHFR mutations, pathology and platinum response: using a two-way table of association using Pearson’s chi-squared significance and Fisher’s exact test for association of each MTHFR mutation on blood pathology and platinum response.

MTHFR Allele	Total no. of Mutations	Serum Folate	Serum Vitamin B12	Active B12	Homocysteine	Platinum Resistant	Partial Sensitivity	Platinum Sensitive
Homozygous C677T	1	P = 0.185	1 in range P = 0.890	P = 0.055	P = 0.940	P = 0.610	P = 0.706	P = 0.444
		FE = 0.682	FE = 1.000	FE = 0.591	FE = 1.000	FE = 1.000	FE = 1.000	FE = 1.000
Homozygous A1298C	2	P = 0.185	2 in range P = 0.783	P = 0.590	P = 0.661	P = 0.461	P = 0.586	P = 0.269
		FE = 0.463	FE = 1.000	FE = 0.844	FE = 0.983	FE = 1.000		FE = 0.520
Heterozygous C677T	3	P = 0.500	3 in range P = 0.680	P = 0.343	P = 0.284	P = 0.538	P = 0.495	P = 0.918
		FE = 0.829	FE = 1.000	FE = 0.642	FE = 0.553	FE = 0.504	FE = 1.000	FE = 1.000
Heterozygous A1298C	7	P = 0.480	6 in range 1 over range P = 0.783	P = 0.548	P = 0.284	P = 0.504	P = 0.826	P = 0.656
		FE = 0.872	FE = 1.000	FE = 1.000	FE = 0.292	FE = 0.597	FE = 1.000	FE = 0.673
Heterozygous C677T + A1298C	5	P = 0.320	1 deficient 4 in range P = 0.117	P = 0.202	P = 0.285	P = 1.000	P = 0.538	P = 0.211
		FE = 0.379	FE = 0.326	FE = 0.293	FE = 0.388	FE = 1.000	FE = 0.504	FE = 0.312
No mutation	4	P = 0.185	2 in range 2 over range P = 0.062	P = 0.565	P = 0.284	P = 0.275	P = 0.420	P = 0.102
		FE = 0.217	FE = 0.135	FE = 0.833	FE = 0.460	FE = 0.549	FE = 1.000	FE = 0.260

**Table 6:** MTHFR mutations and dietary intakes: Using a two-way table of association using Pearson’s chi-squared significance and Fisher’s exact test for association of each MTHFR mutation on dietary intakes of nutrients.

MTHFR Allele	Total No. of Mutations	Dietary Folate Intake		Dietary Vitamin B12		Dietary Vitamin B6		Dietary Zinc	
		Below RDI <400 mcg/d	Above RDI >400 mcg/d	Below RDI <2.4 mcg/d	Above RDI >2.4 mcg/d	Below RDI <1.5 mg/d	Above RDI >1.5 mg/d	Below RDI <8 mg/d	Above RDI >8 mg/d
Homozygous C677T	1	0/6	1/19	1/14	0/11	1/22	0/3	0/8	1/17
		P = 0.566 Fisher’s Exact = 1.000		P = 0.137 Fisher’s Exact = 0.320		P = 0.763 Fisher’s Exact = 1.000		P = 0.484 Fisher’s Exact = 1.000	
Homozygous A1298C	2	1/6	1/19	0/14	2/11	0/22	0/3	1/8	1/17
		P = 0.369 Fisher’s exact = 0.430		P = 0.312 Fisher’s exact = 1.000		P = 0.664 Fisher’s exact = 1.000		P = 0.569 Fisher’s exact = 1.000	
Heterozygous C677T	3	2/6	1/19	3/14	0/11	3/22	0/3	3/8	0/17
		P = 0.065 Fisher’s Exact = 0.133		P = 0.007 Fisher’s exact = 0.024		P = 0.586 Fisher’s exact = 1.000		P = 0.007 Fisher’s exact = 0.024	
Heterozygous A1298C	7	1/6	6/19	1/14	6/11	5/22	2/2	2/8	5/17
		P = 0.478 Fisher’s exact = 0.637		P = 0.236 Fisher’s exact = 0.362		P = 0.018 Fisher’s exact = 0.070		P = 0.819 Fisher’s exact = 1.000	
Heterozygous C677T + A1298C	5								
		0/6	5/19	1/14	4/11	5/22	0/2	1/8	4/17
		P = 0.160 Fisher’s Exact = 0.289		P = 0.520 Fisher’s Exact = 1.000		P = 0.461 Fisher’s exact = 1.000		P = 0.520 Fisher’s exact = 1.000	
No mutation	4	1/6	3/19	0/14	4/11	4/22	0/2	0/8	4/17
		P = 0.959 Fisher’s Exact = 1.000		P = 0.134 Fisher’s Exact = 0.269		P = 0.520 Fisher’s exact = 0.700		P = 0.134 Fisher’s exact = 0.269	

**Table 7:** MTHFR mutation, platinum response, pathology and dietary intakes: multinomial logistic regression (prob >chi2) analysing the association between MTHFR mutations, platinum sensitivity and blood pathology results and dietary intakes.

MTHFR Allele	Total No. of Mutations	Platinum Response	Serum Folate (*p* = )	Active B12 (*p* = )	Homocysteine (*p* = )	Dietary Folate Intake (*p* = )	Dietary Vitamin B12 Intake (*p* = )	Dietary Vitamin B6 Intake (*p* = )	Dietary Zinc Intake (*p* = )
Homozygous C677T	1	Sensitive	0.149	0.4218	0.5844	0.5101	0.1424	0.5918	0.5101
		Partial	0.1772	0.6514	0.7267	0.7142	0.1833	0.7959	0.6734
		Resistant	0.1504	0.6137	0.6552	0.5918	0.2651	0.7551	0.5509
Homozygous A1298C	2	Sensitive	0.2013	0.4384	0.4284	0.2156	0.2096	0.3399	0.2156
		Partial	0.337	0.8168	0.7264	0.4241	0.4441	0.6265	0.5148
		Resistant	0.2233	0.6531	0.5635	0.4524	0.2496	0.5622	0.5151
Heterozygous C677T	3	Sensitive	0.9067	0.4997	0.6010	0.2161	0.0157	0.7643	0.0086
		Partial	0.7221	0.2802	0.6079	0.0790	0.0030	0.4873	0.0030
		Resistant	0.7308	0.4740	0.3925	0.1811	0.0068	0.5884	0.0189
Heterozygous A1298C	7	Sensitive	0.7106	0.4855	0.2326	0.6467	0.3560	0.0597	0.8259
		Partial	0.8094	0.6194	0.1842	0.6571	0.1530	0.0547	0.8925
		Resistant	0.5962	0.5254	0.1996	0.6278	0.4053	0.0610	0.7752
Heterozygous C677T + A1298C	5	Sensitive	0.2902	0.2632	0.1265	0.0627	0.2976	0.2652	0.2145
		Partial	0.5873	0.4457	0.2161	0.0510	0.2657	0.5530	0.2657
		Resistant	0.6793	0.5044	0.8448	0.2097	0.7991	0.6174	0.8006
No mutation	4	Sensitive	0.0478	0.1160	0.0886	0.1275	0.0350	0.1010	0.0514
		Partial	0.2151	0.3066	0.5292	0.5314	0.1797	0.3739	0.1797
		Resistant	0.240	0.1913	0.4013	0.3739	0.0514	0.2972	0.0731

## Data Availability

The data that support the findings of this study are available from the corresponding author upon reasonable request.
